# Dementia post-radiotherapy: improvement with acetylcholinesterase inhibitor. A case report

**DOI:** 10.1590/S1980-57642009DN30100013

**Published:** 2009

**Authors:** Antonio Eduardo Damin, Lílian S. Morillo, Tíbor Rilho Perroco, Wilson Jacob Filho, Cássio Machado de Campos Bottino

**Affiliations:** 1 Behavioral and Cognitive Neurology Unit, Department of Neurology, and Cognitive Disorders Reference Center (CEREDIC).; 2 Hospital das Clínicas of the University of São Paulo, School of Medicine, São Paulo, SP, Brazil.; 3 Disciplina de Geriatria of the Hospital das Clínicas of the University of São Paulo, School of Medicine, São Paulo, SP, Brazil.; 4 Proter - Institute of Psychiatry, Hospital das Clínicas of the University of São Paulo, School of Medicine, São Paulo, SP, Brazil.

**Keywords:** dementia, therapeutics, radiotherapy, cholinesterase inhibitors

## Abstract

Cognitive decline associated with radiotherapy is a progressive complication that affects many patients submitted to this form of treatment. The lack of an effective treatment drives research for new treatment options to improve the quality of life of patients with this disorder. We report the case of a 64 year-old man who developed a severe dementia of the frontal subcortical type, which was associated with subcortical frontal lesions and appeared as a late complication of radiotherapy used to treat a pituitary tumor. After many pharmacological attempts to improve his cognitive and behavioral problems, the patient showed a significant improvement in the cognitive, functional and behavioral impairments after treatment with an acetylcholinesterase inhibitor. This report discusses hypotheses for the positive effect of this treatment.

## Abstract (pt)

A demência associada à radioterapia é uma complicação grave e tardia que acomete muitos pacientes submetidos à RT. A falta de um tratamento efetivo faz com que novas opções de tratamento sejam pesquisadas, no intuito de melhorar a qualidade de vida dos pacientes que apresentam esta complicação. Apresentamos o caso de um homem de 64 anos que anos depois de radioterapia utilizada para tratamento de um tumor de hipófise evoluiu com uma síndrome demencial frontal grave com lesões subcorticais frontais. Depois de muitas tentativas farmacológicas para melhorar seus transtornos cognitivos e comportamentais houve importante melhora cognitiva, funcional e comportamental com o uso de inibidor da acetilcolinesterase. Hipóteses para explicar este efeito positivo do tratamento são discutidas.

The cerebral damage caused by the radiotherapy (RT) is classified traditionally into acute, early delayed and late forms. The acute form usually occurs during the first days after RT and shows symptoms such as fever, headache, nauseas, drowsiness and worsening of focal neurological symptoms. The early delayed form refers to the clinical or radiological worsening which occurs within some weeks or up to a maximum of 12 to 18 months after RT, being responsible for the worsening of edema in brain MRI in up to one-third of gliomas, and in 5 to 20% of meningiomas or cerebral metastasis some months after RT.^1^ In the late forms, there is often progressive and chronic damage of the CNS months or years after RT application. The cerebral damage caused by the RT is dose-dependent. One study showed that patients submitted to a total radiation doses <35 cGy did not show any signs of cognitive impairment, while all the patients with a total irradiation dose >45 cGy showed profound cognitive and behavioral alterations. Those patients who received brain radiation of between 35 and 45 cGy showed slowness of executive function, and profound alterations in frontal functions, such as attentional and judgment impairments and loss of insight, similar to that presented by patients suffering from subcortical vascular dementia.^2^ Among the late forms, we should emphasize the focal cerebral necrosis and the cerebrovascular disease associate with RT. The focal cerebral necrosis of RT occurs after treatment of primary tumors, ^3^ brain metastasis and accidental irradiation of the CNS in pituitary tumors or head and neck tumors.^4^ In anaplastic gliomas among survivors more than 12 months after RT, its incidence is higher than 10%.^4^ The necrosis usually occurs in the proximities of the irradiated tumor^5^ and in pituitary tumors, while the focal necrosis predominates in frontal and temporal lobes and brain stem.^6^ The necrosis can occur from 4 months up to 7 years after RT, peaking between 15–18 months. The treatment is limited: dexametasone produces clinical and radiological improvement, but in most patients the improvement is temporary and these patients become dependent on corticosteroid.^7^ There are anecdotal reports of improvement with warfarin^8^ and hyperbaric oxygen.^9^ With regard to the cerebrovascular manifestations associated with RT, amaurosis fugax, transient ischemic attacks or strokes with intervals from 6 months to more than 20 years after RT (on average 10–20 years) have been described.^10^ Arteriography usually shows vascular disease limited to the irradiated area, with unusual sites of stenosis, such as at the proximal portions of carotids arteries. There is scant available data concerning treatment with endarterectomy, antiplatelet inhibitors or anticoagulants. It is believed that arterial stenosis after radiotherapy is a result of the acceleration of a pre-existing atherosclerosis in the vessels, since most of these patients have dislipidemia.^11, 12^

With regard to treatment for a cognitive impairments following cranial irradiation, there are no proven treatments nor are there any known effective preventive strategies.^13^ One phase II study of donepezil spanning 24 weeks in irradiated brain tumors patients showed significant improvements on tests of attention and concentration, verbal and figural memory, mood and emotional/social/brain aspects, suggesting that patients with longer than 6-month survivals following brain tumors and partial or whole brain irradiation therapy may have neuronal injury with an associated acetylcholine deficiency, and that they can clinically respond to acetylcholinesterase inhibitor.^13^ We report the case of a patient who, after RT for the treatment of a pituitary tumor, developed a progressive and severe dementia that improved with the use of an acetylcholinesterase inhibitor.

## Case report

A 64 year-old man, a retired chemical engineer was evaluated at the Reference Center for Cognitive Disorders of the Hospital das Clínicas da Faculdade de Medicina da Universidade de São Paulo for memory problems and apathy. According to his wife, the symptoms had started 6 years earlier, when the patient had two episodes of topographic disorientation on his way back home from work. The patient was seen by a physician and during the investigation, neuroimaging exams were normal, except for the incidental finding of a pituitary tumor. The patient was submitted to the surgery for removal of the tumor and the pathological examination showed a pituitary adenoma with expression of LH and FSH by immunohistochemistry. The patient was submitted to radiotherapy (RT) at this time, with total dose of 4500 cGy. The patient resumed normal activities at home and work. After 1 year of RT, his wife noticed that the patient initiated slow and progressive difficulty in recognizing his family members, while presenting temporo-spatial disorientation, difficulty to store new information and difficulty in reading and writing. Of special attention, was the report by the wife that the patient had, since the outset, apathy and visual hallucinations with visions of people and animals.

In the ensuing 18 months, there was significant worsening and patient started to get lost even in places close to home and frequently did not recognize his own wife and children, and lately gets lost at home and forgets who he is.

His past medical history included high blood pressure, angioplasty due to coronary hearth disease in 2001, chronic renal failure, dyslipidemia and hypothyroidism in use of levothyroxine 75 mcg/ day. Physical examination was normal. In the first consultation, the patient communicated little, sometimes had severe difficulty remaining awake and had psychomotor agitation. At the neurological examination he showed repetitive speech (repeated the same words several times), gait with a decrease in passive balance of members, bilateral increase in tonus, normal muscle strength and exalted primitive reflexes with snouting, bilateral palmomental and grasping reflexes. The cognitive evaluation yielded a Mini-mental state score of 13/30 (temporal orientation=1, spatial orientation=3, immediate recall=3, attention and calculation=1, recall=0, language=5); CAMCOG of 20 (Orientation: 4/10, Language comprehension: 7/15, Language expression: 3/15, remote memory: 0/6, recent memory: 0/4, learning memory: 1/17, attention: 1/7, Calculation: 2/2, abstract thinking: 1/8, perception: 0/11); the brief battery of cognitive screening with Naming/Perception: 1, incidental memory: 0, immediate memory: 1, late memory of 5 minutes: 0, recognition: 0; and verbal fluency of 1 fruit and 2 animals in 1 minute, and clock drawing test watch of 2.

The evaluation with the caregiver revealed a neuro-psychiatric inventory of 51 (Deliriums 9; Hallucinations 9; Apathy 8; Disinhibition 8; Agitation 6; Depression 6), the Questionnaire of Functional Activity of Pfeffer had a score of 30, while measure on the Zarit scale of caregiver stress was 46 and for Hachinski was 5.

**Figure 1:**
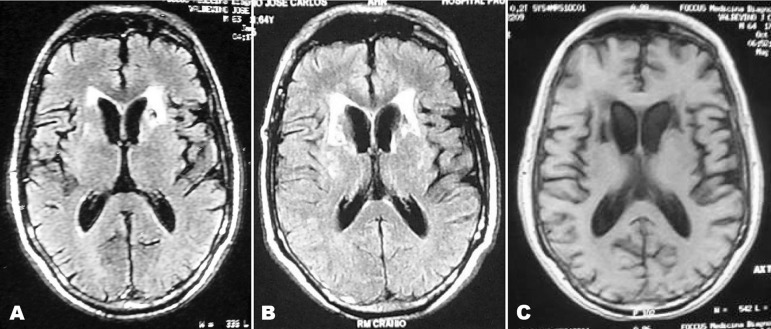
There is a difference of 15 months between image a and b. Note the progressive worsening of the lesions localized in the white matter, especially in the frontal subcortical area.

The patient was normal on laboratory tests for screening dementias and on digital EEG. The examination of cerebrospinal fluid was also normal. Brain MRI showed lesions suggestive of subcortical gliosis and sparse lacunes with frontal predominance without signs of hippocampal atrophy (lesions showed progressive worsening in the successive images, as shown in Figure 1).

On the AngioMRI of cranial vessels, the patient had arterial stenosis of small vessels in segmental branches of anterior cerebral artery with right predominance. Based on the results of imaging exams, the diagnosis of dementia associated with radiotherapy was reached, given focal damage in frontal subcortical area (focal cerebral necrosis), and cerebrovascular disease associated with RT, because of stenosis of vessels in areas close to regions where RT was performed, and stenosis in unusual sites of the cerebral arteries (segmental branches of anterior cerebral artery).

The patient developed worsening of hallucinations with introduction of risperidone up to 6 mg and, later, olanzapine up to 20 mg, with no improvement. Finally, the patient experienced partial improvement with the use of haloperidol, titrated until control of hallucinations and agitation was achieved, but with clear worsening in the pre-existing muscular rigidity. For the nocturnal agitation, clonazepam was tried initially, with worsening of daytime sleepiness. These symptoms showed significant improvement with the introduction of trazodone. Despite the medications used, the patient continued with progressive deterioration in cognitive impairment.

After discussing the case donepezil was introduced, at which point the patient had MMSE=6/30 (temporal orientation=0, spatial orientation=1, immediate recall=3, attention and calculation=1, recall=0, language=1) and CDR=3. In subsequent visits, his wife reported a significant improvement in cognitive impairment, hallucinations and behavioral disturbance. The MMSE score increased to 12 (subtest of MMSE and other cognitive tests not available) after taking 10 mg of donepezil daily. In addition, the patient showed a significant improvement in language, reported by his wife and also detected by clinical evaluation, along with an obvious improvement in his ability to communicate verbally with the medical team. At the time, the patient was more active and demonstrated improvement in locomotion. After two months of reaching the maximum dose of donepezil (10 mg), testosterone and growth hormone deficiency was diagnosed, with somatrotopin and testosterone propionate replacement leading to even greater improvement of cognition and apathy. The patient remained relatively stable for several months but had worsened further in terms of disease evolution. The last cognitive assessment was made in July 2008, with MMSE=5 (temporal orientation=0, spatial orientation=0, immediate recall=3, attention and calculation=, recall=0, language=2) and score on Pfeffer=30.

## Discussion

RT for pituitary adenoma is administered in order to reduce the likelihood of tumour recurrence and is standard treatment following surgical removal in some cases of pituitary adenomas.^14^ In our case, progressive dementia occurred after RT dementia and neuroimaging showed lacunes and gliosis in the frontal subcortical area. There are three possibilities with regard to the mechanisms that caused dementia in this patient: focal cerebral necrosis, cerebrovascular disease associated with RT or most probably, a combination of both. Concerning focal cerebral necrosis, we can explain this by the time of onset of symptoms and the presence of lesions near the site of RT, predominantly in the frontal subcortical area, both being consistent with the diagnosis of focal cerebral necrosis. Regarding cerebrovascular disease, the unusual sites of arterial stenosis (segmental branches of anterior cerebral arteries) and the presence of prior dyslipidemia corroborate this hypothesis. The patient had a history of severe dementia, with severe executive and attentional disorders, as well as severe apathy and cognitive slowness, combined with frontal release signs including grasping and snouting characteristic of frontal dementia. The image exams showed diffuse lesions in frontal subcortical area as a probable etiologic mechanism of dementia, since the location of the lesions was consistent with the cognitive deficit presented by the patient. Associated with the predominance of frontal subcortical cognitive deficits, the images did not show clear hippocampal or temporal lobe atrophy, making the diagnosis of Alzheimer’s disease less likely, where Alzheimer’s disease could explain the initial symptom of spatial disorientation. However, no progressive worsening of this spatial disorientation was observed and cognitive symptoms only occurred 1 year after radiotherapy. The hypothesis that spatial disorientation was a result of a stroke which may have occurred at the time of symptom should not be ruled out, although no ischemic lesions were observed on the neurologic images taken at the time. In the light of the data above, and given the possibility of dementia with a vascular component and the known response of vascular dementia to acetylcholinesterase inhibitors, it was decided to introduce a drug with this mechanism of action.

Another factor supporting the introduction of acetylcholinesterase inhibitor was the anatomy of the projections of the cholinergic system from the basal forebrain, especially the basal nucleus of Meynert, mainly through its medial pathway which radiates to the medial orbitofrontal cortex, as well as its lateral pathway which radiates to wide parts of the neocortex, particularly the frontal ramification which in turn radiates to the inferior frontal cortex.^15^

Thus, interruption of these pathways by the damage found in brain white matter, suggesting that a cholinergic deficit could explain the patient’s cognitive impairment. Moreover, the fact that substantial initial improvement was observed in the patient following the introduction of acetylcholinesterase inhibitor, not explained by other mechanisms, corroborated the cholinergic hypothesis. Therefore, it is important to emphasize that, even in cases where no effective treatment is available, as in cases of dementia associated with radiotherapy, trying approaches based on theoretical knowledge of pathophysiology may be valid and discussed by doctors in order to improve the quality of life of patients.
